# The Few, the Strong: Rat Cortex Features Small Numbers of Powerful Connections

**DOI:** 10.1371/journal.pbio.0030111

**Published:** 2005-03-01

How is the brain wired up? Each neuron may connect with hundreds or even thousands of others, and the human brain has a hundred billion neurons. Determining the connection diagram for a whole brain is a truly daunting prospect, and currently well beyond reach. But one way into this thicket is to look for patterns in a small region. In this issue, Dmitri Chklovskii and colleagues show that in the rat visual cortex, some kinds of connection patterns are much more common and much stronger than chance would predict.

To determine the pattern of connections, the researchers placed electrodes into randomly chosen quartets of neurons near each other. They stimulated each in turn, and determined which members responded, and how strongly. Sampling over 800 such quartets, they found 931 actual connections out of a possible 8,050, for an average rate of connectivity of 11.6%. From the group of connected neurons, they then asked about reciprocal connections: what was the likelihood that, if A stimulated B, B stimulated A as well? They found that bidirectionally connected cells were four times as common as expected by chance, a pattern previously observed in other regions of cortex. They asked the same question for groups of three cells, for which there are 16 possible connection patterns. Two patterns stood out as especially significant: (1) A and B talk back and forth with each other, and both listen to C; and (2) A, B, and C all talk with one another. For four cells, although the numbers were too small for statistical analysis, a common over-represented class was chain connections, a kind of a path connecting all four cells that can be drawn without lifting the pencil from the page.[Fig pbio-0030111-g001]

**Figure pbio-0030111-g001:**
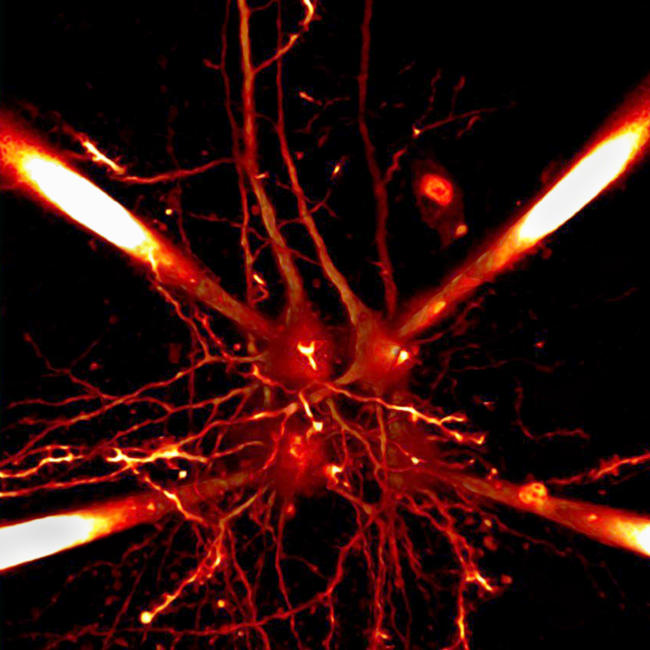
Recording multiple neurons simultaneously

Because the strength with which one neuron stimulates another can be just as important to network function as whether a connection exists at all, the authors examined connection strength as well. They found that connection strengths are distributed broadly, with some connections ten times stronger than the average connection and the strongest 17% of connections contributing half of total synaptic strength. They found that, on average, connections that were part of bidirectional pairs were about 50% stronger than unidirectional ones, and because of this, despite being fewer in number, they disproportionately contributed to the total amount of excitation in the neural network. A similar pattern was found for neuronal triplets—the most highly connected groups of neurons had the strongest connections among them.

Taken together, these results show that neural networks, at least in this portion of the rat brain, are characterized by a vocal minority of unexpectedly strong and reliable connections amidst a large number of weak ones, which suggests the strong ones may play more central roles in local computation or communication. This stands in strong contrast to the usual starting assumption of neural modelers, that connectivity is random. The exact pattern of connectivity seen here for excitatory neurons in one cortical layer (layer 5) may not be universal, and indeed, different patterns have been described in the cerebellum. Nonetheless, the essential feature seen here—“a skeleton of stronger connections in a sea of weaker ones,” as the authors put it—may be an important and common functional feature of brain wiring.

